# Applicability of Precision Medicine Approaches to Managing Hypertension in Rural Populations

**DOI:** 10.3390/jpm8020016

**Published:** 2018-04-30

**Authors:** Jacqueline R. Halladay, Kaitlin C. Lenhart, Kimberly Robasky, Wendell Jones, Wayne F. Homan, Doyle M. Cummings, Crystal W. Cené, Alan L. Hinderliter, Cassandra L. Miller, Katrina E. Donahue, Beverly A. Garcia, Thomas C. Keyserling, Alice S. Ammerman, Cam Patterson, Darren A. DeWalt, Larry F. Johnston, Monte S. Willis, Jonathan C. Schisler

**Affiliations:** 1 Department of Family Medicine, The University of North Carolina at Chapel Hill, Chapel Hill, NC 27599, USA; jacqueline_halladay@med.unc.edu (J.R.H.); wayne.homan@gmail.com (W.F.H.); katrina_donahue@med.unc.edu (K.E.D.); 2 Cecil R. Sheps Center for Health Services Research, The University of North Carolina at Chapel Hill, Chapel Hill, NC 27599, USA; 3 McAllister Heart Institute at The University of North Carolina at Chapel Hill, Chapel Hill, NC 27599, USA; kaitlin.lenhart@gmail.com (K.C.L.); monte_willis@med.unc.edu (M.S.W.); 4 Q2 Solutions|EA Genomics, Morrisville, NC 27560, USA; krobasky@email.unc.edu (K.R.); Wendell.Jones@q2labsolutions.com (W.J.); 5 Department of Family Medicine, East Carolina University, Greenville, NC 27834, USA; cummingsd@ecu.edu; 6 Department of Medicine, The University of North Carolina at Chapel Hill, Chapel Hill, NC 27599, USA; crystal_cene@med.unc.edu (C.W.C.); alan_hinderliter@med.unc.edu (A.L.H.); Thomas_Keyserling@med.unc.edu (T.C.K.); darren_dewalt@med.unc.edu (D.A.D.); 7 Center for Health Promotion and Disease Prevention at The University of North Carolina at Chapel Hill, Chapel Hill, NC 27599, USA; cassiem@email.unc.edu (C.L.M.); beverly_garcia@unc.edu (B.A.G.); larry_johnston@unc.edu (L.F.J.); 8 Department of Nutrition, Gillings School of Global Public Health at The University of North Carolina at Chapel Hill, Chapel Hill, NC 27599, USA; alice_ammerman@unc.edu; 9 Presbyterian Hospital/Weill-Cornell Medical Center, New York, NY 10065, USA; cpatters@nyp.org; 10 Department of Pharmacology and Department of Pathology and Lab Medicine, The University of North Carolina at Chapel Hill, Chapel Hill, NC 27599, USA

**Keywords:** hypertension, GWAS, precision medicine, rural population, SNP-age interaction

## Abstract

As part of the Heart Healthy Lenoir Project, we developed a practice level intervention to improve blood pressure control. The goal of this study was: (i) to determine if single nucleotide polymorphisms (SNPs) that associate with blood pressure variation, identified in large studies, are applicable to blood pressure control in subjects from a rural population; (ii) to measure the association of these SNPs with subjects’ responsiveness to the hypertension intervention; and (iii) to identify other SNPs that may help understand patient-specific responses to an intervention. We used a combination of candidate SNPs and genome-wide analyses to test associations with either baseline systolic blood pressure (SBP) or change in systolic blood pressure one year after the intervention in two genetically defined ancestral groups: African Americans (AA) and Caucasian Americans (CAU). Of the 48 candidate SNPs, 13 SNPs associated with baseline SBP in our study; however, one candidate SNP, rs592582, also associated with a change in SBP after one year. Using our study data, we identified 4 and 15 additional loci that associated with a change in SBP in the AA and CAU groups, respectively. Our analysis of gene-age interactions identified genotypes associated with SBP improvement within different age groups of our populations. Moreover, our integrative analysis identified *AQP4-AS1* and *PADI2* as genes whose expression levels may contribute to the pleiotropy of complex traits involved in cardiovascular health and blood pressure regulation in response to an intervention targeting hypertension. In conclusion, the identification of SNPs associated with the success of a hypertension treatment intervention suggests that genetic factors in combination with age may contribute to an individual’s success in lowering SBP. If these findings prove to be applicable to other populations, the use of this genetic variation in making patient-specific interventions may help providers with making decisions to improve patient outcomes. Further investigation is required to determine the role of this genetic variance with respect to the management of hypertension such that more precise treatment recommendations may be made in the future as part of personalized medicine.

## 1. Introduction

Hypertension (HTN) and its end-organ manifestations including stroke, coronary heart disease, and chronic renal failure are major contributors to morbidity and mortality in the United States and globally [1,2]. On average, life expectancy is reduced by five years among those with HTN, which is responsible for nearly one in every eight deaths worldwide [3]. Multiple important individual, societal, and environmental variables contribute to an individual’s risk of developing HTN [4]. Particularly noteworthy is the persistence of racial disparities in HTN prevalence, control, and untoward outcomes between African Americans (AA) and Caucasians (CAU), despite the fact that a higher proportion of AAs are both aware of and receive treatment for HTN [5].

Typical intervention strategies used to reduce blood pressure (BP) include implementing strategies at various levels of patient influence (patient, family, healthcare provider, community level) [6] and in some cases implementing strategies to enhance control among specific groups, such as African Americans [7,8]. Such interventions aim to reduce BP by improving medication adherence, guiding better lifestyle choices, using home BP monitors, addressing clinical inertia in intensifying anti-hypertensive treatment, using team-based approaches to improve HTN management, and other strategies [7,8,9,10,11].

Additional factors of interest in the study of HTN include advancing our understanding of how genes associate with both the presence of HTN and the responsiveness to interventions aiming to reduce BP, and how genes interact with the many other contributing factors, such as advancing age, that influence the prevalence of HTN. A better understanding of these genetic influences may inform the implementation of targeted and personalized therapies that mitigate the untoward consequences of sustained HTN. 

Genome-wide association studies (GWAS) identified associations between specific genetic loci, mapped by the presence of single nucleotide polymorphisms (SNPs) that represent genetic variation among populations, and the prevalence of HTN [12,13,14,15,16,17,18,19]. Remarkably, Simino and colleagues [12] developed a unique approach to analyzing cross-sectional GWAS data by stratifying hypertension-SNP association data into age brackets, which provided results suggesting that some SNPs associate with BP, but the magnitude and direction of this association varied by age. However, it is not clear whether these data, often obtained from large studies of well-defined populations near major medical institutions, are applicable to subjects in rural areas that often suffer from health disparities. Moreover, it is not known whether these data are germane given the multifactorial nature and numerous different environmental modifiers that interact with genes to influence BP [20,21], a phenomenon seen with other chronic diseases as well [22,23,24]. The multifactorial nature of chronic diseases distinguishes them and their study from Mendelian diseases, such as cystic fibrosis, sickle cell anemia, phenylketonuria, and others, where the presence of specific risk alleles are sufficient to cause a disease phenotype [25,26,27].

Our team developed and implemented a two-year multi-level intervention, called the Heart Healthy Lenoir (HHL) project, to improve clinical management of HTN, with a specific focus on reducing racial disparities in BP levels. The primary outcome of the intervention was the change in systolic blood pressure (SBP) from baseline to 12-month follow-up (hereafter denoted by ΔSBP, calculated as follow-up minus baseline). Five hundred and twenty-five participants with a clinical diagnosis of uncontrolled hypertension participated in the HHL high BP study. We recruited patients whose last recorded SBP was ≥150 mmHg in order to enhance the probability of the subjects having uncontrolled HTN (SBP ≥ 140 mmHg or diastolic blood pressure (DBP) ≥ 90 mmHg) at their study enrollment visit. Along with baseline survey and biometric data, participants were invited to provide blood samples for genetic analyses [28]. Our goal was to determine if precision medicine approaches in a rural population can provide insight into BP regulation and possible responsiveness to a hypertension intervention.

## 2. Materials and Methods

### 2.1. Clinical Approach

#### 2.1.1. Description of High Blood Pressure Study

Details of the design, setting, participants, and implementation of the study are described in Halladay et al. [29] and Cene et al. [30]. Briefly, we conducted a prospective cohort intervention study using a community-based participatory approach that included input from a community advisory committee and the staff at local practices to help inform the intervention content and delivery. Our cohort consisted of 525 English-speaking patients enrolled from six local practices with an established clinical diagnosis of uncontrolled hypertension and an office SBP of ≥150 mmHg during a one-year time frame before enrollment. Our multi-component office-based HTN improvement intervention included strategies at both the practice/organization level (e.g., design team calls, dinner meetings, practice facilitation, and review of electronic health record data) and at the patient level (e.g., telephone coaching, home BP monitoring). The telephone coaching part of our intervention was informed by components of Bosworth’s Take Care of Your Blood Pressure study [31]. We provided practices with an anti-hypertensive medication algorithm based upon guidance included in the Seventh Report of the Joint National Committee on Prevention, Detection, Evaluation, and Treatment of High BP (JNC-7) treatment algorithm to which providers, staff, and patients could refer at the point of care [1].

#### 2.1.2. Description of Lifestyle Study

Two hundred participants in the hypertension intervention were co-enrolled in the HHL lifestyle study. Detailed information on the study design and methods are published [32]. Briefly, the lifestyle study began with a four-month intervention focused on improving dietary fat and carbohydrate quality and increasing physical activity. Over the next eight months, participants received a lifestyle maintenance intervention or could elect to receive a weight loss intervention if their body mass index (BMI) was ≥25 kg/m^2^. Patients could thus be in either study or co-enrolled in both. Hypertension was not a requirement for participation in the lifestyle study.

#### 2.1.3. Study Measures

Baseline and follow-up data were collected as described [29,30,32,33,34]. Blood pressure was measured by trained research staff using the Omron HEM-907 automated BP monitor (Omron Healthcare, Inc., Vernon Hills, IL, USA). A research assistant recorded the average of three sequential measurements obtained at 60-s intervals and followed JNC-7 guidelines for accurate measurement technique [1].

### 2.2. Genomic and Experimental Analyses

#### 2.2.1. DNA Isolation, Purification, and Quality Control

DNA was purified from a total of 512 HHL participants (see Figure S1 for study participation information) with an automated system (Autopure LS, Autogen, Holliston, MA, USA). The DNA samples were quantified in multi-spectral optical density spectrophotometers (SpectraMax Plus, Molecular Devices, San Jose, CA, USA) at two dilutions in duplicate. Fifteen percent of all DNA samples were run on agarose gels for quality assurance verification. Final dilutions of DNA (75 ng/µL) used for genotyping were confirmed using PicoGreen double stranded DNA quantification (Promega, Madison, WI, USA).

#### 2.2.2. Genotyping on the Illumina Platform

Genotypes were generated from genomic DNA using the Infinium workflow, reagents, equipment, and software (Illumina, San Diego, CA, USA) essentially as described by the manufacturer. DNA was amplified, fragmented, precipitated with isopropanol, and resuspended prior to hybridization onto BeadChips containing 50mer probes. After hybridization, enzymatic single base extension with fluorescently labeled nucleotides was conducted to distinguish alleles. Hybridized BeadChips were imaged using an Illumina iScan to determine intensities for each probe. Corresponding genotypes were extracted from intensity data and called using a standard cluster file within Illumina Genome Studio software. A Minimal Information About a Microarray Experiment (MIAME) compliant dataset of the microarray data generated is available at the NCBI database of Genotypes and Phenotypes (dbGaP, study ID phs001471).

After Genome Studio calls were made, the quality of the genotype calls was reviewed in detail, examining SNPs with low call rates, SNPs that violated Hardy-Weinberg equilibrium assumptions, and SNPs that putatively had no variation. After review and correction using segmented population-based custom clustering, low call rate SNPs were reduced by 10%, and >2000 SNPs with no apparent variation were adjusted, sometimes manually, to reflect actual population diversity (Figure S2). The remaining ~175k SNPs with no variation were removed from the study and other SNPs failing the initial Hardy-Weinberg equilibrium (HWE) testing were re-clustered to a state that met HWE assumptions, amounting to approximately 3000 and 1000 SNPs in the AA and CAU ancestral cohorts, respectively (Table S1). 

#### 2.2.3. SNP-Level Analysis of Admixture and Relatedness

We excluded SNPs that were less than 80% present across all samples or had fewer than 1.6% heterozygous calls. Starting with all 512 samples and including data from four HapMap samples of known population origin, we applied principal components analysis to determine the genetic population groups and to flag samples with admixture. We also used this analysis to identify pairs of samples with 68% or more SNP similarity (near relatives) as both admixture and related individuals can obfuscate GWAS results. In the case of related individuals, we included only the subject with the largest absolute ΔSBP for data analysis.

#### 2.2.4. Imputing Single Nucleotide Polymorphisms

All HHL DNA samples identified as either AA (305) or CAU (199) were imputed for a total of 504 imputed samples. The array data were exported into plink format and converted into chromosome-specific variant call format, applying the following filters: merge replicate probes, switch the alternate (ALT) or reference (REF) sequence if deemed necessary by reference, exclude markers where neither REF nor ALT matches the reference, exclude markers where REF is not AGCT. Additionally, in preparation for Beagle the following filters were further applied: remove markers not in the reference, fill ALT values in from reference where genotype is entirely homozygous for reference.

Samples were imputed twice, once with the Michigan imputation server (using Minimac v2013.7.17 [35] and once with Beagle v4.1 [36]. All 504 samples imputed with Beagle were run against the 2504-sample reference panel from 1000 genomes. The Haplotype Reference Consortium (HRC, 65k haplotypes) reference panel was used to run the CAU samples on the Michigan imputation server, and the Consortium on Asthma among African-ancestry Populations in the Americas (CAAPA) reference panel was used to run the AA samples on the imputation server. A brief summary of coverage regarding the panels and how they performed with the target marker set (the markers from the genotyping array) is provided (Table S2). However, the Illumina genotyping arrays are sparse compared to the reference panels. We filtered our array data for conformity and the markers remaining used for the variant calls are indicated (Table S3).

#### 2.2.5. Pre-Modeling Activities

We performed statistical analyses to measure the association of demographic and clinical variables to the ΔSBP, stratified by ancestry, using a bivariate linear fit or one-way analysis for continuous variables or categorical variables, respectively (JMP Pro, v12.1, SAS, Cary, NC, USA). We then generated a multivariable linear model to test the effect of variables in the presence of other clinically relevant variables and their potential association with ΔSBP (JMP Pro, v12.1) where *β*_0_ is the *y*-intercept and *β*_n_ represents the standardized beta coefficient of the effect of each variable:

(1) $$\Delta \mathrm{SBP}=\beta_{0}+(\beta_{1}\cdot \mathrm{Age})+(\beta_{2}\cdot \mathrm{Gender})+(\beta_{3}\cdot \mathrm{EverSmoked})+(\beta_{4}\cdot \mathrm{Diabetes})+(\beta_{5}\cdot \mathrm{LS})+(\beta_{6}\cdot \%\mathrm{weightloss})+(\beta_{7}\cdot \mathrm{BMI}).$$

#### 2.2.6. Pre-Modeling Activities

We filtered SNPs to include only those with minor allele frequencies >5% and with 100% call-rates. Next, each SNP was tested for association with baseline SBP or ΔSBP using multivariable linear regression within each ancestral cohort. The first model accounted for age, gender, and smoking including interaction terms with age. HetSNP and HomSNP correspond to the heterozygous and homozygous status of the SNP:

(2) $$\mathrm{SBP}\;\mathrm{or}\;\Delta \mathrm{SBP}=\beta_{0}+(\beta_{1}\cdot \mathrm{Age})+(\beta_{2}\cdot \mathrm{Gender})+(\beta_{3}\cdot \mathrm{EverSmoked})+(\beta_{4}\cdot \mathrm{Gender}\cup \mathrm{Age})+(\beta_{5}\cdot \mathrm{EverSmoked}\cup \mathrm{Age})+(\beta_{6_{1}}\cdot \mathrm{HetSNP})+(\beta_{6_{2}}\cdot \mathrm{HomSNP})+(\beta_{7_{1}}\cdot \mathrm{HetSNP}\cup \mathrm{Age})+(\beta_{7_{2}}\cdot \mathrm{HomSNP}\cup \mathrm{Age}),$$

A second model used for ΔSBP included a variable to account for co-participation in the lifestyle intervention:

(3) $$\mathrm{SBP}\;\mathrm{or}\;\Delta \mathrm{SBP}=\beta_{0}+(\beta_{1}\cdot \mathrm{Age})+(\beta_{2}\cdot \mathrm{Gender})+(\beta_{3}\cdot \mathrm{EverSmoked})+(\beta_{4}\cdot \mathrm{Gender}\cup \mathrm{Age})+(\beta_{5}\cdot \mathrm{EverSmoked}\cup \mathrm{Age})+(\beta_{6_{1}}\cdot \mathrm{HetSNP})+(\beta_{6_{2}}\cdot \mathrm{HomSNP})+(\beta_{7_{1}}\cdot \mathrm{HetSNP}\cup \mathrm{Age})+(\beta_{7_{2}}\cdot \mathrm{HomSNP}\cup \mathrm{Age})+(\beta_{8}\cdot \mathrm{Lifestyle}),$$

#### 2.2.7. Baseline Systolic Blood Pressure Association Testing

Risk SNP genotypes were obtained from the microarray data where available. Where not available, risk SNP genotypes were imputed genotypes from the CAAPA and HRC panels available on the Michigan Imputation server, according to the ancestry determined for the sample. Where genotypes were not available on the Michigan Server panel, the genotype was obtained from the Beagle imputation against the 1000 genomes panel. Specifically, three risk SNPs were imputed with Beagle: rs12408339, rs17428471, rs4373814. We used Equation (3) to determine *p* values for SNP associations with baseline SBP. 

### 2.3. Human Studies

This study was approved by the Institutional Review Board at the University of North Carolina at Chapel Hill with data collected from September 2011 to November 2014 and registered as # NCT01433484 at clinicaltrials.gov. All study participants gave verbal consent for administration of the study screening questionnaire (to assess eligibility) and written consent before study data were collected. 

## 3. Results

### 3.1. Study Population 

#### 3.1.1. Genetic Ancestry of the Study Population

**Figure 1:**
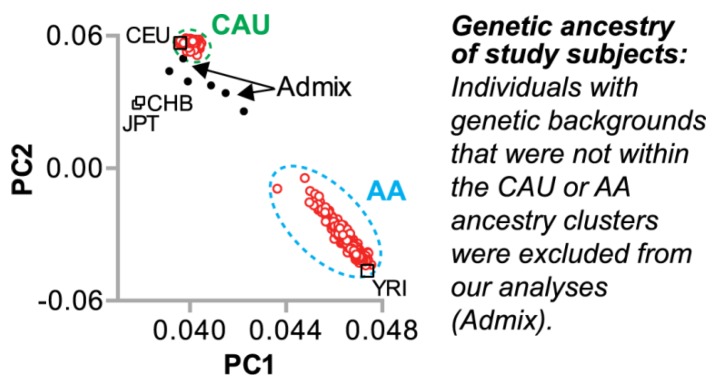
Principal component analysis of all genotyped Heart Healthy Lenoir (HHL) study participants. Five hundred and twelve HHL samples identified with either Caucasian American (CAU) or African American (AA) ancestry (○) or admixed samples (●). HapMap samples of known ancestral origins are identified (□): CEU, Utah residents with Northern and Western European ancestry; CHB, Han Chinese in Beijing, China; JPT, Japanese in Tokyo, Japan; YRI, Yoruba in Ibadan, Nigeria. PC1: principal component 1; PC2: principal component 2.

We evaluated 512 genetic samples obtained from the HHL cohort by principal components analysis using over 700,000 SNPs (Figures S1 and S2, Table S1) to identify subjects of either African or European ancestry (Figure 1) as well as relatedness. We then removed subjects with admixture to identify a subset of genetically unrelated subjects assigned to the office-based HTN improvement intervention who had BP measurements at baseline and 12-month follow-up, referred to hereafter as the “HTN cohort,” as well as a smaller cohort of HHL subjects that did not receive the HTN intervention (Table S4). The HTN cohort was stratified by genetic ancestry into two groups comprised of 193 subjects of AA ancestry and 123 subjects of Northern and Western European (Caucasian American, CAU) ancestry, respectively (Figure S1).

#### 3.1.2. Clinical Characteristics of the Study Population

The AA subjects included in this study were younger, had a greater mean body mass index (BMI), and higher mean diastolic BP without differences in mean systolic BP compared to the CAU subjects (Table 1). The AA group also had higher rates of diabetes, higher high-density lipoprotein cholesterol (HDL-C) values, and were more likely to be prescribed anti-hypertensives from different classes of anti-hypertensive medications compared to the CAU group.

### 3.2. Identifying Clinically Relevant Variables that Associate with Systolic Blood Pressure

#### Bivariate and Multivariable Analyses of Clinical Variable Associations with ΔSBP over One Year

**Figure 2:**
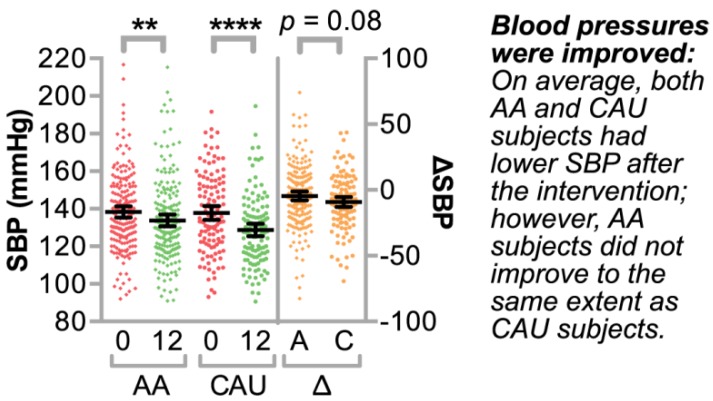
Systolic blood pressures of participants enrolled in hypertension intervention. The systolic blood pressures (SBP, left *y*-axis) of African American (AA, A, *n* = 193) or Caucasian (CAU, C, *n* = 123) participants in the hypertension intervention at the start of the intervention (0) and after 12 months of the intervention as well as the change in SBP (Δ, right *y*-axis) after 12 months are represented by a dot plot and summarized by mean ±95% confidence intervals: **** *p* < 0.0001 and ** *p* < 0.01 via the paired *t*-test of SBP. The *p* value of the unpaired *t*-test comparing the Δ of AA versus CAU cohorts is also indicated.

Overall, the intervention was successful in lowering SBP in both AA and CAU groups, with mean ΔSBPs (± standard deviations) of −4.6 ± 23.7 and −9.2 ± 20.5 mmHg in the AA and CAU participants, respectively (Figure 2). We performed a bivariate analysis of variables of interest with ΔSBP (Table 2). The sign of the estimated beta in this analysis reflects either an increase (+) or decrease (−) in SBP over one year relative to the variable of interest. We identified a negative association between ΔSBP and age in both groups, meaning that older participants had a greater decrease in SBP. We also observed opposite associations between ΔSBP and smoking status in our groups, with smoking associated with either an increase or decrease in SBP after one year of the intervention in AA or CAU groups, respectively. Additionally, we identified associations with lifestyle (LS) co-participation and weight loss in the AA group, suggesting that LS co-participation may have contributed to the ΔSBP in this group. Although there were differences in both the percentage of subjects taking anti-hypertensives as well as in the number of classes anti-hypertensives prescribed comparing the AA and CAU groups (Table 1), within the two groups we did not find any association between ΔSBP and either the number of different classes of anti-hypertensives or the total number of anti-hypertensives that study subjects were initially prescribed (Figure S3a–d). Moreover, when we considered all subjects (AA or CAU) that were prescribed only a single class of medication, there was no difference in ΔSBP (Figure S3e). Overall, these data suggest that age, smoking, and LS co-participation are trait variables of interest with respect to ΔSBP. 

Although bivariate analysis provides some insight into factors that may influence SBP, this approach does not control for the confounding effects of multiple variables on BP regulation. Therefore, we performed a multivariable analysis. In Table 3, we show results for our multivariable linear model for ΔSBP, Equation (1). Interestingly, in this model only the effect of age in both AA and CAU groups and LS co-participation in the AA group had associations with ΔSBP at *p* < 0.05. The effect of weight loss did not impact ΔSBP in this model, likely due to the association of weight loss with LS co-participation (*p* = 0.028); therefore, we opted to use the LS variable in lieu of weight loss in subsequent models. 

### 3.3. Association Analysis of Candidate Blood Pressure Polymorphisms in a Rural Population

Our multivariable analysis informed the generation of two equations to test the association of the SNP main effect (either heterozygous or homozygous status) in the context of other variables identified in Table 3 (age), as well as historically relevant variables associated with BP control such as gender [37] and smoking status [20]. Given the strong effect of age, we also included interactions terms for age and each variable. Equation (2) was used to test the association of SNPs with SBP or ΔSBP levels. Equation (3) included an additional term to control for co-participation in the LS intervention and was used to test the association of SNPs with ΔSBP.

For precision medicine to impact population health, we must consider if and how data, such as genetic variation, can be used as potential risk indicators for both populations and individual patients. Several large GWAS identified risk SNP variants associate with BP [18,19,38,39]. Traditionally, GWAS are performed on large and affluent patient populations, primarily of European ancestry. One of our prime objectives was to determine whether BP risk SNPs from the aforementioned studies apply to our study population. Therefore, we tested the association of baseline SBP with the 19 and 29 previously identified risk SNPs from AA and CAU subjects, respectively. Remarkably, we replicated 13 of these associations within our HTN cohort, including the main SNP effect as well as with the SNP-age interaction variable (Table 4, Table S5). In fact, four loci originally identified in European cohorts also associated with baseline SBP in our AA subjects. Given that these variants associate with HTN and other cardiovascular comorbidities, we hypothesized that these same SNPs would also associate with the change in SBP after one year of our intervention, as the same genetic influences of BP regulation may also contribute to how individuals respond to BP lowering interventions. Next, we tested the risk SNPs for their association with ΔSBP, performing a similar association analysis. Only one (rs592582) of the 13 SNPs that associated with baseline SBP also associated with ΔSBP (Table 4, Table S6). These results suggest that variation associated with hypertension may be distinct from variation associated with responsiveness to HTN interventions.

### 3.4. Genome-Wide Association Analysis of Genetic Variation with ΔSBP

#### 3.4.1. Single Nucleotide Polymorphism Associations with ΔSBP

**Figure 3:**
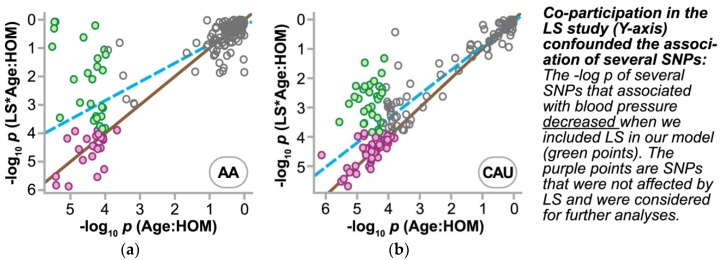
Lifestyle co-participation correction on SNP discovery. The effect of including a lifestyle co-participation variable (LS) in the model for ΔSBP is represented by a scatter plot of the *p* values of the Age:HOM interaction term with or without the LS variable on either the *y*- or *x*-axis, respectively, in the AA (**a**) or CAU (**b**) cohort. Regression analysis (dashed line) indicates the overall effect of the correction by how far it deviates from no change (solid line). Individual SNPs that passed the discovery cutoff of *p* < 1 × 10^−4^ with the LS correction or SNPs that were confounded by LS participation and excluded from additional analyses are indicated by either magenta- or green-filled points, respectively. Open points represent additional SNPs with no association to ΔSBP (*p* > 1 × 10^−4^). HOM: homozygous status of the SNP.

**Figure 4:**
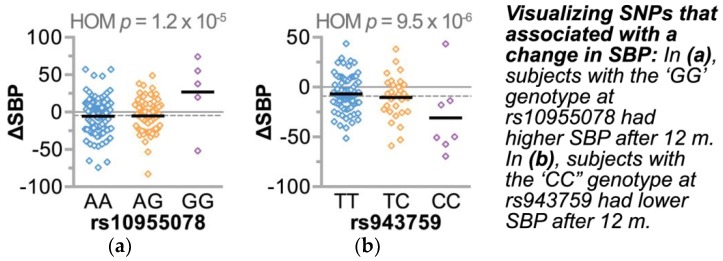
The SNP main effect associated with a change in blood pressure after 12 months of the intervention. The change in SBP (ΔSBP) after 12 months of the intervention in either (**a**) AA or (**b**) CAU study participants (*n* = 193 and 123, respectively), represented by a dot plot and summarized by the median. The *p* value of the association of the HomSNP variable (HOM) with ΔSBP for each SNP is indicated. The dashed line represents the mean ΔSBP for each cohort.

We extended our ΔSBP association analyses to other regions in the genome using our HHL cohort to identify other SNPs that associate with the responsiveness of our intervention. Approximately 585,000 probes were mapped to unique SNPs and matched to *p* values produced by our linear model that adjusted for age, gender, and smoking using Equation 2. The quantile-quantile probability (Q-Q) plots revealed that no single SNP reached genome-wide significance at *p* < 1 × 10^−8^, which was not surprising given the complex phenotype of BP regulation and the size of our study. However, the Q-Q plots were skewed in *p* values for the homozygous term and the homozygous:age interaction term starting at observed *p* values less than 1 × 10^−4^, and hence we restricted our analyses to SNPs in these regions (Figure S4). Knowing the confounding effect of LS co-participation in our model of ΔSBP (Table 3), we first tested the impact of the LS correction term in our multivariable model. To account for the LS effect, we included an additional 58 and 22 subjects to the AA and CAU groups that were only exposed to the LS intervention (and not the HTN intervention, Table S4). We compared the *p* values of the SNP main effect and SNP-age interaction with and without the LS variable in the model, Equations 2 and 3. This allowed us to identify SNPs confounded by LS co-participation (Figure 3, Figure S5). Additionally, consistent with our multivariable analysis, the inclusion of the LS variable had a larger effect in the AA group compared with the CAU group. The increased effect size in the AA group was likely due to the higher baseline SBP in the co-enrolled AA patients compared to the CAU patients (142.6 and 139.5 mmHg, respectively). This approach allowed us to identify 26 and 74 candidate SNPs (*p* range 1 × 10^−4^ – 1.2 × 10^−6^) that associated with ΔSBP and were unaffected by LS co-participation in the AA and CAU groups, respectively (Figure 4, Tables S7 and S8). 

#### 3.4.2. Refining SNPs of Interest and Identifying SNP-Age Interactions

**Figure 5:**
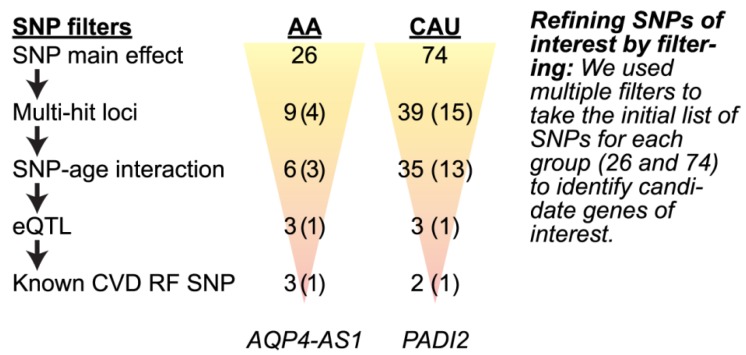
Workflow to identify genetic regions of interest that associate with ΔSBP. We used a series of data filters (SNP filters) to refine potential loci associated with the responsiveness to our HTN intervention. The number of individual SNPs associated with ΔSBP within each ancestral group is indicated (SNP main effect) and the remaining number of SNPs after each filter (**↓**) as well as the corresponding number of loci represented by the SNPs are provided in parentheses: #SNPs (#loci). eQTL: expression quantitative trait loci; CVD RF: cardiovascular disease risk factor; *AQP4-AS1*: Aquaporin 4 antisense RNA 1; *PADI2*: protein-arginine deiminase type II.

**Figure 6:**
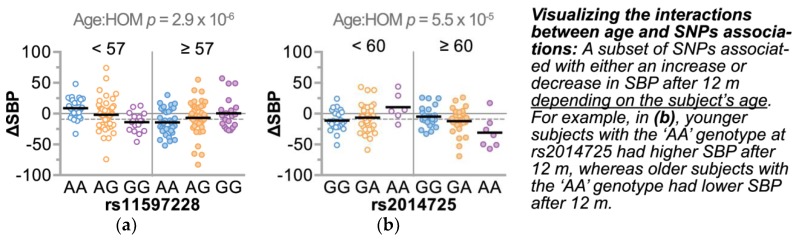
SNP–age interactions associated with a change in blood pressure after 12 months of the intervention. The change in SBP (ΔSBP) after 12 months of the intervention in either (**a**) AA or (**b**) CAU study participants (*n* = 193 and 123, respectively), represented by dot plot and summarized by the median. To demonstrate the SNP-age interaction, each cohort was stratified over the median age. The *p* value of the association of the interaction between age and the HomSNP variable (Age:HOM) with ΔSBP for each SNP is indicated. The dashed line represents the mean ΔSBP for each cohort.

Given the discovery nature of our study and the cohort design of the HTN intervention, we applied a workflow to filter, refine, and validate candidate SNPs (Figure 5). First, we identified genomic regions represented by multiple SNP associations to ΔSBP, resulting in four and 15 loci in the AA and CAU groups, respectively (Table 5 and Table 6). Consistent with the SNP-age associations seen with baseline SBP, three of the four loci from the AA group and 13 of the 15 loci from the CAU group also had SNP-age interactions with ΔSBP (Figure 5). Given the recent observation of gene–age interactions on BP [12] and our results reported here, we further explored the gene-age observation by stratifying each ancestral group by median age. Using this approach, we identified SNPs that associated with either a successful or unsuccessful intervention (mean ΔSBP < 0, or ≥ 0, respectively) depending on age (Figure 6), suggesting these gene-age interactions may have influenced a participant’s response to the HTN intervention.

#### 3.4.3. Single Nucleotide Polymorphisms Associate with Changes in Gene Expression and Other Cardiovascular Risk Factors

**Figure 7:**
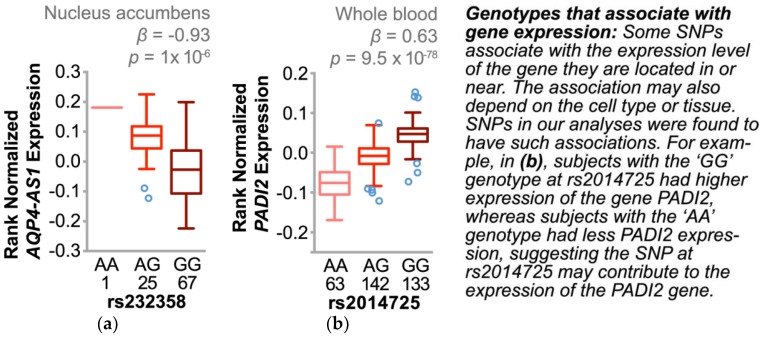
Expression of quantitative trait loci involving candidate SNPs associated with ΔSBP. Cis eQTL analysis of the eSNP rs232358 and rs2014725 with the number of subjects, genotype, and corresponding expression levels of either (**a**) *AQP4-AS1* or (**b**) *PADI2* is represented by boxplots (5–95% confidence intervals) with outliers identified (open circles). The *p* value and beta coefficient (β) of the linear regression are noted.

Whereas SNP associations can inform us of possible heritable linkages to disease, integrating data from association studies with expression quantitative trait locus (eQTL) studies was shown by us [40,41] and others [42] to help identify genes that may play functional roles in a complex trait such as BP regulation. Therefore, we identified the multi-hit loci that also associated with a change in tissue-specific gene expression using well-defined, independent samples (range 93–338 samples, false discovery rate < 1%) [43]. We identified a single locus in each cohort comprised of three eSNPs (expression SNP) in perfect linkage disequilibrium in the AA cohort (*AQP4-AS1*, *p* = 1 × 10^−6^) as well as three eSNPs in high linkage disequilibrium (*R*^2^ > 0.97) in the CAU cohort (*PADI2*, *p* range = 1.1 × 10^−4^ to 2.8 × 10^−101^). *AQP4-AS1* is an uncharacterized gene encoding a long non-coding RNA of unknown function. The expression of *AQP4-AS1* is restricted to specific regions of the brain. Interestingly, the only tissue with a corresponding eQTL for *AQP4-AS1* is in the nucleus accumbens region of the brain. Neurons in the nucleus accumbens are involved in inhibiting fight-or-flight responsive BP increases [44]. Moreover, in a rodent chronic hypertension model, animals with high BP had a reduction in both dendritic spine density and length in neurons within the nucleus accumbens, a pathology that worsened with age [45]. On the other hand, *PADI2* is more ubiquitously expressed throughout the body with the highest levels in whole blood, skeletal muscle, and the spinal cord. We observed robust eQTLs across multiple tissues (Table S9), with the strongest eQTL found in whole blood (Figure 7). *PADI2* encodes an enzyme involved in protein citrullination, a clinically targeted pathway implicated in a range of diseases such as atherosclerosis, rheumatoid arthritis, lupus, and multiple sclerosis [46]. 

Finally, given the limited size of our study, the numerous risk factors that contribute to BP regulation, and the sparsity of association between previously identified BP SNPs and ΔSBP (Table 4), we leveraged the power of larger GWAS studies to determine if our candidate loci associate with other cardiovascular disease risk factors. We validated our candidate SNPs in larger cohorts [47] to broadly look at cardiovascular risk factor associations with our candidate SNPs. Interestingly, the three *AQP4-AS1* SNPs associated with BMI (*p* range 0.011–0.013) [48] whereas rs737428 and rs2014725 in *PADI2* associated with either low-density lipoprotein cholesterol (*p* = 0.017) [49] or total cholesterol (*p* = 0.047) [50]. These data suggest that these loci may be involved in processes important to cardiovascular health and responsiveness to HTN interventions.

## 4. Discussion

We explored associations of SNPs and BP change in a cohort of African American and Caucasian participants (Figure 1) during a one-year multi-level intervention aimed to reduce BP in patients with established HTN in Eastern, NC (USA) (Figure 2). Remarkably, within our small, rural population of study participants in a region of the country that suffers disproportionally from higher cardiovascular disease risk, we associated several known genetic variants with baseline SBP levels (Table 4) by controlling for age, gender, and smoking. Moreover, the SNP rs592582 also associated with the responsiveness to the intervention. The minor allele of rs592582 associated with higher baseline SBP and lower SBP after one year, suggesting that this SNP not only associates with the presence of hypertension, but also associates with responsiveness to interventions like those employed in the HHL study. The remaining candidate SNPs that associated with baseline SBP did not associate with the responsiveness to the intervention, which prompted us to perform an unbiased genome-wide association analysis of SNPs with ∆SBP in combination with exclusionary data filtering (Figure 5) to identify other genetic factors that may contribute to the intervention response. We identified four and 15 loci in either our AA or CAU groups that were identified by multiple SNPs with either SNP main effects and/or SNP–age interactions that associated with a change in SBP (Figure 4, Table 5 and Table 6). Additionally, we explored the interaction of participant age and SNPs to evaluate the potential impact of age on the responsiveness to the intervention within a SNP group. Several of these loci identified genotypes in which the BP increases or decreases after one year of the intervention depended on the age group of the participants (Figure 6, Table 5 and Table 6). Our observation of gene–age interactions with BP is consistent with other recent studies that also observed an influence of age on the association of SNPs with cardiovascular risk factors such as SBP or BMI [12,51]. Hence, the impact of age on SNP associations with the responsiveness to our HTN intervention may provide further insight into the underlying biology of a why certain individuals respond differently to hypertension treatments and the possible influence of age on the effectiveness of the intervention. 

Our approach led us to two genes of interest, *AQP4-AS1* and *PADI2*. These two genes and the specific variants we identified also associated with other important cardiovascular disease risk factors such as BMI and blood lipids in other independent cohorts [48,49], suggesting that these loci play a role in cardiovascular health risk. Additionally, these SNPs associated strongly with the expression of the gene where they are located (Figure 7), identifying these as eSNPs and perhaps indicating a functional role for these variants in our interventional response. *AQP-AS1* is a long non-coding RNA comprised of 10 overlapping non-coding RNA (ncRNA) transcripts of unknown function conserved in both mice and zebrafish [52,53]. *PADI2*, however, is known to encode a peptidyl arginine deiminase that catalyzes the post-translational deimination of proteins by converting arginine residues into citrullines, including myelin basic protein, vimentin, actin, and histones [54]. The eSNPs we identified associated with a robust change in *PADI2* expression in multiple tissues including blood cells, heart, aorta, and both visceral and subcutaneous adipose (Table S9). Altered PADI2 activity is implicated in neurodegenerative and inflammatory diseases [54] and most recently, vascular angiogenesis [55]. *PADI2* expression and anti-citrullinated protein antibodies were higher in smokers, suggesting that increased *PADI2* expression, particularly in genetically susceptible subjects (Figure 7b), promotes more robust pathophysiological responses to environmental stressors [56,57,58]. Our data suggest that these *PADI2* eSNPs and the expression of *PADI2* may be have differential effects on blood pressure regulation depending on age (Figure 6b) and may be useful in understanding an individual’s response to blood pressure interventions, particularly in smokers.

Other dietary supplement studies and medication studies evaluated SNP associations with BP change in Han Chinese cohorts. Gu and Kelly tested the effectiveness of sodium and potassium supplementation, respectively, on a subsample of hypertensive patients that were part of the Genetic Epidemiology Network of Salt Sensitivity Study [37,59]. Gu et al. [37] examined associations between 11 renin-angiotensin-aldosterone-system candidate genes with SBP, DBP, and mean arterial BP change among 1860 Han Chinese subjects who either had HTN or were the sibling, offspring, or spouse of the individuals with HTN. This cohort consumed a low sodium diet for seven days followed by a high sodium diet for an additional seven days. Five SNPs were independently associated with BP responses to a low sodium diet, while just one was associated with BP response to a high sodium diet. They also shared findings of two additional SNPs that were significantly associated with BP reduction in only males who were exposed to the low sodium diet. The investigators suggested that these genes may play a critical role in the salt sensitivity of BP and could help identify patients that may benefit most from a low sodium diet. Furthermore, using participants from this same GenSalt study, Kelley et al. [59] performed a separate analysis on 1906 study subjects that were exposed to a high sodium diet for 14 days, but were additionally provided 60 mmols of potassium daily for the last seven days. Their results identified regions on chromosomes 3 and 11 that may harbor susceptibility loci for dietary potassium sensitivity and a novel variant in the angiotensin II receptor suggested to be a strong predictor of BP response to potassium sensitivity. As in Gu’s work, they suggest that such findings may provide insights into the pathophysiology of hypertension and the genetic mechanisms that underlie potassium sensitivity. They too concluded that the ultimate value of these types of discoveries might be in guiding people with specific genotypes to dietary interventions that may provide the greatest impact on BP control.

Multiple anti-hypertensive medication trials have been performed to attempt to identify SNPs associated with responsiveness to individual classes of anti-hypertensives [60,61]. Additional studies identified SNPs associated with opposite effects on a subject’s BP with different classes of anti-hypertensive medications [62]. For example, some SNPs are associated with a BP reduction in response to one class of medication (e.g., angiotensin receptor blockers) and a BP increase in response to other classes (e.g., diuretics). The results from Turner et al. [62] and our study presented here further the call to develop personalized medicine approaches in treating patients with hypertension, allowing personal (genetic- and age-based) recommendations for specific combinations of drugs. As in our study, few findings in the these aforementioned medication studies reached the traditional level of statistical significance deemed sufficient in GWAS [63]; however, many of the SNPs identified in these studies and ours are potentially important to both disease etiology and tailoring treatments for HTN.

Limitations: As a cohort study reporting pre-post measures, we cannot rule out the possibility that the observed changes in BP may be due to secular trends or other factors that were not captured in our data collection and were not instigated by the multi-level intervention per se. The pragmatic nature of implementing this kind of an intervention with research-naïve clinical partners was both a limitation and a strength [29]. We designed and implemented the intervention with broad stakeholder input to maximize feasibility and sustainability, but in a multi-level intervention in “real world” practices, there is no way to disentangle the effect of any particular aspect of the intervention as being more or less important in BP control. Likewise, we did not have a measure of medication adherence beyond self-report, an important component that needs to be addressed for a broad range of diseases if we are to move forward with precision medicine approaches to care. Our research teams and practicing clinicians are keenly interested in including anti-hypertensive medication metabolite data in both trials and routine care as a measure of patient compliance and adherence. This approach could reliably identify which anti-hypertensive medications are being taken by individual patients. We did not find any associations between different measures of medication exposure and the effectiveness of the intervention. However, designing studies that focus both on health disparities and specific anti-hypertensives may identify gene-drug interactions that could ultimately aid in using genetic data as a component of blood pressure care.

Additionally, we tested for genetic variation associations using a platform ensuring broad coverage across the genome that captures variation in both of our ancestral groups, but certainly there could be additional, important genetic variation that contributed to the responsiveness to the intervention that was not represented on our arrays or imputation panels. Due to the small sample size, we used an exploratory *p* value to establish significance in this study. Additionally, for our modeling work, not all variables retained in our multiple regression model were independently associated with the outcome within each race. We included variables noted in the literature to be associated with BP outcomes in other papers, but in some cases from very different populations [20,37,64]. Without the data of prior studies on populations, such as that we had in the HHL study, we decided to include the variables listed. Lastly, our study population was from a small region in Eastern NC. Thus, our results should not be generalized to larger populations. However, the genetic ancestry of our study population is reflective of study populations from large US-based GWAS studies; hence, we believe that our population is reasonably representative of the larger African American and Caucasian population in the US.

## 5. Conclusions

Our results support the concept that genetic variation data from large association studies can be utilized at a local, practice-based level to help identify genetic risk for HTN. Furthermore, by measuring individual responses to HTN interventions, we can start to identify genetic variants and other important factors identified in our study, such as age, that could ultimately be used to guide treatments. Implementing more HTN interventions that include genomic analyses across multiple locales and communities will allow us to determine the impact and utility of precision medicine on directing treatments for HTN. We encourage investigators to continue to find solutions for the numerous influences on patients with HTN that will allow us to reduce the untoward effects on the families and communities affected by this disease. 

## Figures and Tables

**Table 1:** Baseline characteristics and ancestral cohort differences of subjects enrolled in the hypertension intervention.

	AA	CAU	*p*
**Demographics**			
Number of genotyped participants	193	123	
* Age at enrollment, mean (range)	57 (24–92)	60 (25–91)	0.0103
Male sex, *n* (%)	60 (31)	41 (33)	0.7113
*** Education: HS or less, *n* (%)	152 (79)	74 (60)	0.0005
*** Low literacy^†^, *n* (%)	52 (29)	12 (10)	0.0002
Employed full or part time, *n* (%)	71 (37)	47 (38)	0.8124
*** Household income ≤ $40,000 *n* (%), (missing = 14%)	148 (90)	70 (65)	0.0001
* Currently have health insurance, *n* (%)	135 (70)	99 (80)	0.0480
Self-rated health good-excellent, *n* (%)	118 (61)	75 (61)	1.0000
Co-enrollment in lifestyle study, *n* (%)	84 (44)	43 (35)	0.1579
**Cardiovascular Disease Risk Factors**			
Current cigarette smoker, *n* (%)	44 (23)	27 (22)	0.8909
* Diabetes (self-report or HbA1c ≥ 6.5), *n* (%)	94 (49)	42 (34)	0.0143
Total cholesterol (mg/dL), mean (SE)	186 (3.0)	194 (3.4)	0.1715
*** HDL-C (mg/dL), mean (SE)	53 (1.0)	47 (1.3)	0.0003
Systolic BP (mmHg), mean (SE)	138 (1.5)	138 (1.9)	0.8523
* Diastolic BP (mmHg), mean (SE)	83 (1.0)	80 (1.1)	0.0310
Systolic BP ≥140 mmHg, *n* (%)	84 (44)	54 (44)	1.0000
**Physiologic Factors**			
* Weight (kg), mean (SE)	101 (1.7)	95 (2.4)	0.0307
* Body Mass Index, mean (SE)	37 (0.7)	35 (0.9)	0.0214
Number of comorbidities, mean (SE)	3.4 (0.1)	3.7 (0.2)	0.2956
** Glomerular Filtration Rate (mg/dL), mean (SE)	88 (1.7)	81 (1.7)	0.0045
Medication and Adherence			
*** Taking BP lowering medication, *n* (%)	182 (94)	100 (81)	0.0006
*** Number of BP medication classes, mean (SE)	2.1 (0.1)	1.6 (0.1)	0.0010

Data presented as mean (standard error) or count (proportion): *, **, *** correspond to *p* < 0.05, 0.01, 0.001, respectively, via *t*-test for continuous data or Fisher’s exact test for categorical data between ancestral cohorts. ^†^ Low literacy determined by scoring under 23 using the Short Test of Functional Literacy in Adults. BP: blood pressure; HDL-C: high-density lipoprotein cholesterol; SE: standard error.

**Table 2:** Bivariate analysis of ΔSBP and trait variables. The indicated traits were analyzed for association with ΔSBP within each ancestral cohort.

	AA	CAU
Trait	Estimate (SE)	Estimate (SE)
Age (years)	−0.43 (0.14) **	−0.37 (0.18) *
Lifestyle participation (no)	6.51 (3.42) ^‡^	1.77 (3.90)
BMI (per unit)	−0.17 (0.19)	0.34 (0.18) ^‡^
Smoking (some vs. none)	13.68 (8.60)	−24.67 (11.87) *
Smoking (some vs. daily)	15.30 (9.26) ^‡^	−24.67 (12.40) *
Smoking (none vs. daily)	1.61 (4.40)	0.00 (4.62)
Diabetes (no)	−2.95 (3.42)	−5.77 (3.87)
Gender (male)	2.31 (3.70)	3.15 (3.92)
Weight loss (per percent)	−0.66 (0.33) *	−0.06 (0.29)

Results are reported as the estimate (*β* or mean difference for continuous or nominal variables, respectively) and standard error (SE); ^‡^, *, ** indicate *p* ≤ 0.10, 0.05, 0.01, respectively.

**Table 3:** Multivariable regression analysis of ΔSBP and traits of interest. The indicated traits were analyzed for association with ΔSBP in a multivariable linear model within each ancestral cohort.

	AA	CAU
Trait	Estimate (SE)	Estimate (SE)
Age (years)	−0.59 (0.15) ***	−0.38 (0.19) *
Lifestyle participation (no)	3.49 (1.73) *	0.91 (1.84)
BMI (per unit)	−0.34 (0.21)	0.19 (0.25)
Smoking history (ever)	−2.09 (2.14)	−3.89 (2.41)
Diabetes (no)	−2.52 (1.72)	−1.84 (2.11)
Gender (male)	0.03 (1.90)	2.11 (1.91)
Weight loss (per percent)	−0.39 (0.33)	−0.16 (0.29)

Results are reported as the estimate (*β* or mean difference for continuous or nominal variables, respectively) and standard error (SE); *, *** indicate *p* ≤ 0.05, 0.001, respectively.

**Table 4:** Associations of previously identified risk single nucleotide polymorphisms (SNPs) with either baseline SBP or ΔSBP.

dbSNP ID	Gene(s)	Chr	GRCh37	Cohort	β HET	β HOM	β Age: HET	β Age: HOM
rs592582	*XR_001739753*	2	157773386	AA	↑ [↓]		↓ [↑]	
rs243601	*C21orf91-OT1*	21	19159766	AA	↑		↓	
rs243603	*C21orf91-OT1*	21	19160300	AA	↑		↓	
rs243605	*C21orf91-OT1, C21orf91*	21	19161120	AA	↑		↓	
rs243607	*C21orf91-OT1, C21orf91*	21	19161515	AA	↑		↓	
rs2220511	*C21orf91-OT1, C21orf91*	21	19164911	AA	↑		↓	
rs2258119	*C21orf91*	21	19167479	AA		↓		↑
rs1799945	*HFE*	6	26091179	AA ^†^	↓		↑	
rs381815	*PLEKHA7*	11	16902268	AA ^†^	↓	↑	↑	↓
rs3184504	*SH2B3*	12	111884608	AA ^†^		↑		↓
rs2521501	*FES*	15	91437388	AA ^†^	↓		↑	
rs17477177	*CTB-30L5.1*	7	106411858	CAU		↑		↓
rs1378942	*CSK*	15	75077367	CAU	↓		↑	

The Single Nucleotide Polymorphism Database identifier (dbSNP ID) from the National Center for Biotechnology Information, associated gene, chromosome (chr), position in GRCh37, and the HTN ancestral group of SNP main effects and SNP-age interactions that associated with baseline SBP are indicated by arrows. Associations of either the heterozygous (HET) or homozygous (HOM) genotype with ΔSBP are indicated by arrows in brackets []. The arrows indicate either positive (↑) or negative (↓) estimates (β) of the SNP main effect or the SNP-age interaction term on SBP (or ΔSBP) at p < 0.05. SNPs identified initially in CAU populations that associated with SBP in our AA cohort are indicated (^†^).

**Table 5:** Loci with multiple SNPs that associate with ΔSBP in the AA cohort. The dbSNP identifier (ID), description of region, associated gene, chromosome (chr), and position in GRCh37 of the SNP main effects and SNP-age interactions that associated with ΔSBP are indicated by arrows.

dbSNP ID	Region	Gene(s)	Chr	GRCh37	*β* HET	*β* HOM	*β* Age: HET	*β* Age: HOM
rs16942954	intronic	*AQP4-AS1, CHST9*	18	24501350		↑		↓
rs16942955	intronic	*AQP4-AS1, CHST9*	18	24502493		↑		↓
rs232358	intronic, 3’ UTR	*AQP4-AS1, CHST9*	18	24492099	↓			
rs380625	intronic, 3’ UTR	*AQP4-AS1, CHST9*	18	24493117	↓			
rs1181704	intronic, 3’ UTR	*AQP4-AS1, CHST9*	18	24492641	↓			
rs11597228	intergenic	*CELF2*	10	10660838		↓		↑
rs4747873	intergenic	*CELF2*	10	10686085		↓		↑
rs7906433	intergenic	*KLF6*	10	3888845	↓	↑	↑	↓
rs12255472	intergenic	*KLF6*	10	4466023		↑		↓

The arrows indicate either positive (↑) or negative (↓) estimates (*β*) of the SNP or SNP-age interaction on ΔSBP at *p* < 1 × 10^−4^.

**Table 6:** Loci with multiple SNPs that associate with ΔSBP in the CAU cohort. The dbSNP identifier (ID), description of region, associated gene, chromosome (chr), and position in GRCh37 of the SNP main effects and SNP-age interactions that associated with ΔSBP are indicated by arrows.

dbSNP ID	Region	Gene(s)	Chr	GRCh37	*β* HET	*β* HOM	*β* Age: HET	*β* Age: HOM
rs2014725	intronic	*PADI2*	1	17417253		↓		↑
rs2235910	intronic	*PADI2*	1	17425829		↓		↑
rs737428	intronic	*PADI2*	1	17429185		↓		↑
rs4949959	intergenic	*RWDD3*	1	95766707	↓	↑	↑	↑
rs4950044	intergenic	*RWDD3*	1	95766797	↓	↑	↑	↓
rs6683355	intergenic	*RWDD3*	1	95773106	↓	↑	↑	↓
rs7519220	intergenic	*SRP9, ENAH*	1	225863345		↓		↑
rs7365361	intergenic	*SRP9, ENAH*	1	225864622		↓		↑
rs943759	intergenic	*LOC102723834*	1	225886318		↓		↑
rs6576973	3’ UTR	*ARID5A*	2	97218367	↓		↑	
rs7608325	intergenic	*KANSL3*	2	97305080			↑	
rs7690085	intronic	*FSTL5*	4	162709000	↑	↓	↓	
rs13130537	intronic	*FSTL5*	4	162718372	↓	↓	↑	↑
rs10026821	intronic	*SORBS2*	4	186540503	↑		↓	
rs10030246	intronic	*SORBS2*	4	186541887	↑		↓	
rs37957	intronic	*LOC100505921, LOC105375139*	7	8000971		↓		
rs37968	intronic	*LOC100505921, LOC105375139*	7	8005973				↓
rs1468594	intronic	*GLCCI1*	7	8122313	↓	↑	↑	↓
rs10966220	intergenic	*IZUMO3, ELAVL2*	9	24113158		↓		
rs10812027	intergenic	*IZUMO3, ELAVL2*	9	24113936		↓		
rs10886170	intergenic	*GHITM, NRG3*	10	85091864		↓		↑
rs10886214	intergenic	*GHITM, NRG3*	10	85127739	↓	↑	↑	↓
rs11244854	intronic	*ADAM12*	10	127850629				↓
rs1674927	intronic	*ADAM12*	10	127852395				↓
rs7337547	intergenic	*SPRYD7, KPNA3*	13	50443527	↓			
rs11617754	intronic	*SPRYD7*	13	50501980	↑			
rs9805613	intergenic	*SLITRK6*	13	86982353	↑		↓	
rs9302073	intergenic	*SLITRK6*	13	87002553		↑		↑
rs8021103	intronic	*LOC105370510*	14	56177363	↑	↓	↓	↑
rs10498477	intronic	*LOC105370510*	14	56180099	↑	↑	↓	↓
rs2134919	intergenic	*EXOC5, OTX2*	14	57412758	↑	↓	↓	↑
rs6573129	intergenic	*EXOC5, OTX2*	14	57648321		↓		↑
rs7158266	intergenic	*EXOC5, OTX2*	14	57648751		↓		↑
rs10136042	intergenic	*EXOC5, OTX2*	14	57665761		↑	↑	↓
rs10135064	intergenic	*EXOC5*	14	57668859		↓		↑
rs3742578	missense, 3’ UTR	*EXOC5*	14	57672715	↓	↓	↓	↑
rs7141911	3’ UTR	*EXOC5*	14	57672871		↓		↑
rs198480	intergenic	*CTNNBL1, BLCAP*	20	36280827		↑		↓
rs1928630	intergenic	*CTNNBL1, BLCAP*	20	36286035		↑	↑	↓

The arrows indicate either positive (↑) or negative (↓) estimates (*β*) of the SNP or SNP-age interaction on ΔSBP at *p* < 1 × 10^−4^.

## Supplementary Materials

The following are available online at http://www.mdpi.com/2075-4426/8/2/16/s1, Figure S1: Summary of sample call rates prior to and post data-driven and custom analysis of SNPs, Figure S2: HHL stratification of intervention participation, Figure S3: Association between anti-hypertensives and the change in systolic blood pressure, Figure S4: Q-Q plots of the SNP main effect and SNP-age interaction effect, Figure S5: Lifestyle co-participation correction on SNP discovery, Table S1: Improvement in calls for autosomal SNPs, Table S2: Loci information for imputation, Table S3: Imputed targets, Table S4: Baseline characteristics of participants enrolled solely in the lifestyle intervention, Table S5: Baseline SBP risk SNP hits extended data, Table S6: One-year ΔSBP risk SNP hits-extended data, Table S7: One-year ΔSBP hits, extended AA cohort data, Table S6: One-year ΔSBP hits, extended CAU cohort data, Table S9: eQTL analysis of SNPs of interest.
