# Supplementary material for: Applicability of Precision Medicine Approaches to Managing Hypertension in Rural Populations
Source: J Pers Med. 2018 Apr 30;8(2):16. doi: 10.3390/jpm8020016 (PMC6023309; doi:10.3390/jpm8020016)
Supplement: Supplementary file 1 [file jpm-08-00016-s001.zip › 2018-03-23 HTN genes SNPs Supplementary Figs for resub.pdf]

Supplementary Figures

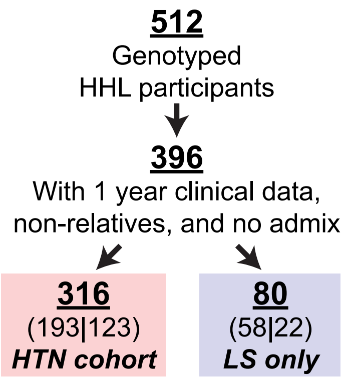

Figure S1. HHL stratification of intervention participation. A total of 512 HHL participants were genotyped. After one year, 396 subjects remained to be used for SNP association analyses. 316 subjects received the HTN intervention, making up the HTN cohort described in this report. 80 subjects did not receive the HTN intervention and only participated in the lifestyle (LS) intervention, referred to as the LS only cohort. The number of AA vs CAU subjects in each cohort is indicated (AA|CAU).

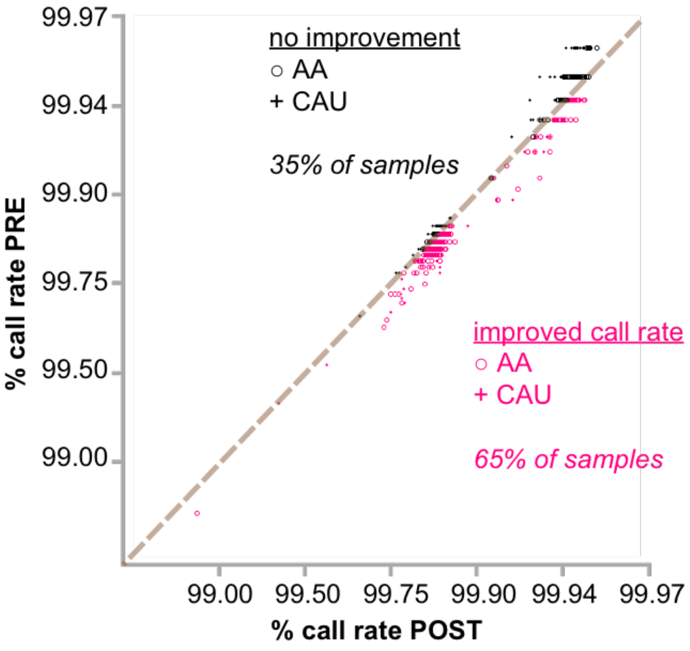

Figure S2. Summary of sample call rates prior to and post data-driven and custom analysis of SNPs. Samples in magenta (65%) experienced an increase in Call Rate due to the data-driven custom analysis. Circle (○) or plus (+) symbols indicate Americans of either African (AA) or European (CAU) descent. The AA subjects benefited more from customer clustering than persons of European descent.

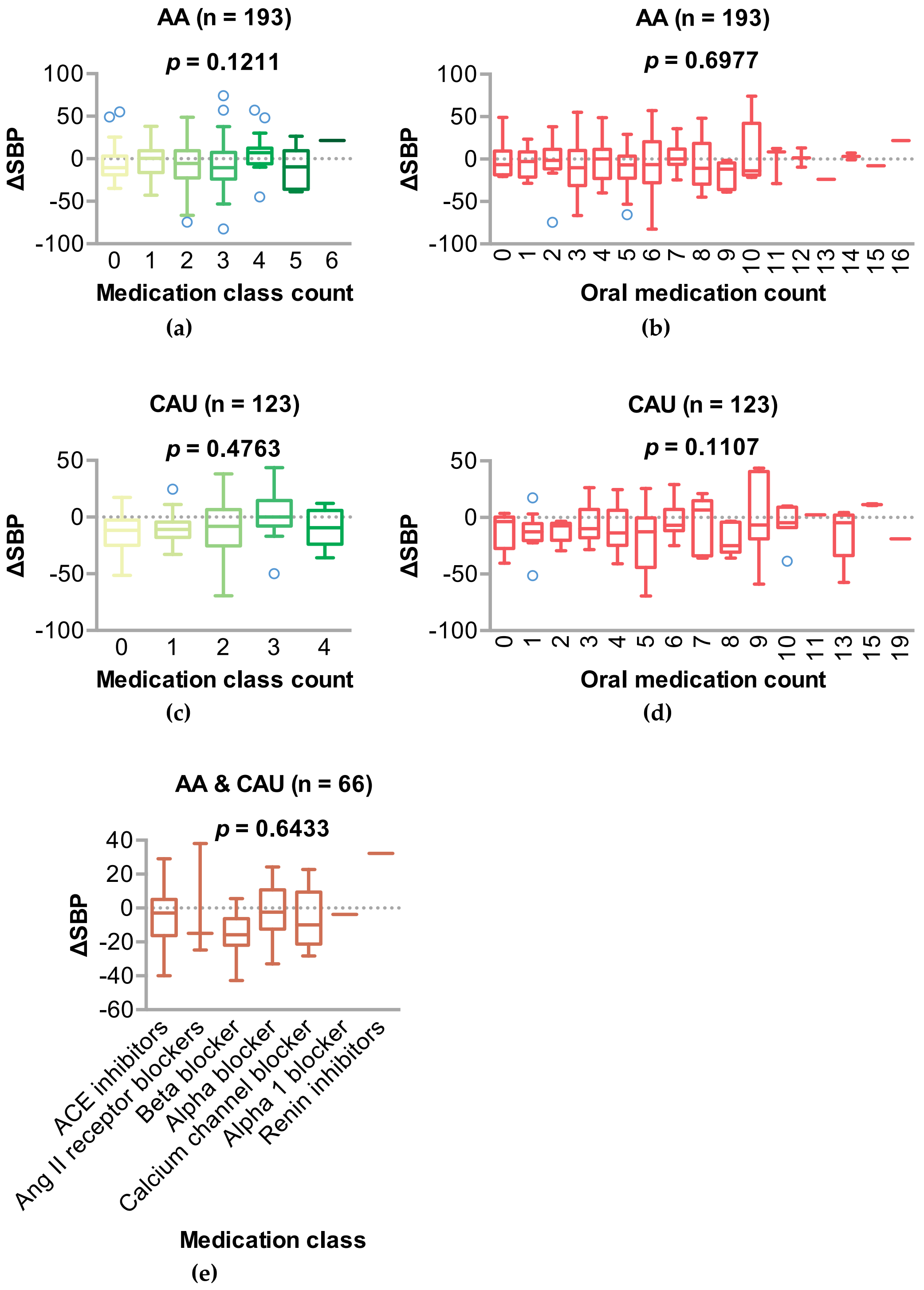

Figure S3. Association between anti-hypertensives and the change in systolic blood pressure. Kruskal-Wallis analysis (ANOVA on ranks) of the association between ΔSBP and either the number of different classes of anti-hypertensives **(a)** and **(c)**, or the total number of oral anti-hypertensives regardless of class, **(b)** and **(d)** that study participants were prescribed in either the AA **(a)** and **(b)** or CAU **(c)** and **(d)** groups. A subset of subjects from both groups that were prescribed only a single anti-hypertensive medication was analyzed via ANOVA for the association between ΔSBP and specific classes of anti-hypertensives. Data are represented by box-whisker plots (Tukey) with outliers indicated (○), corresponding *p* value of the statistical test, and N.

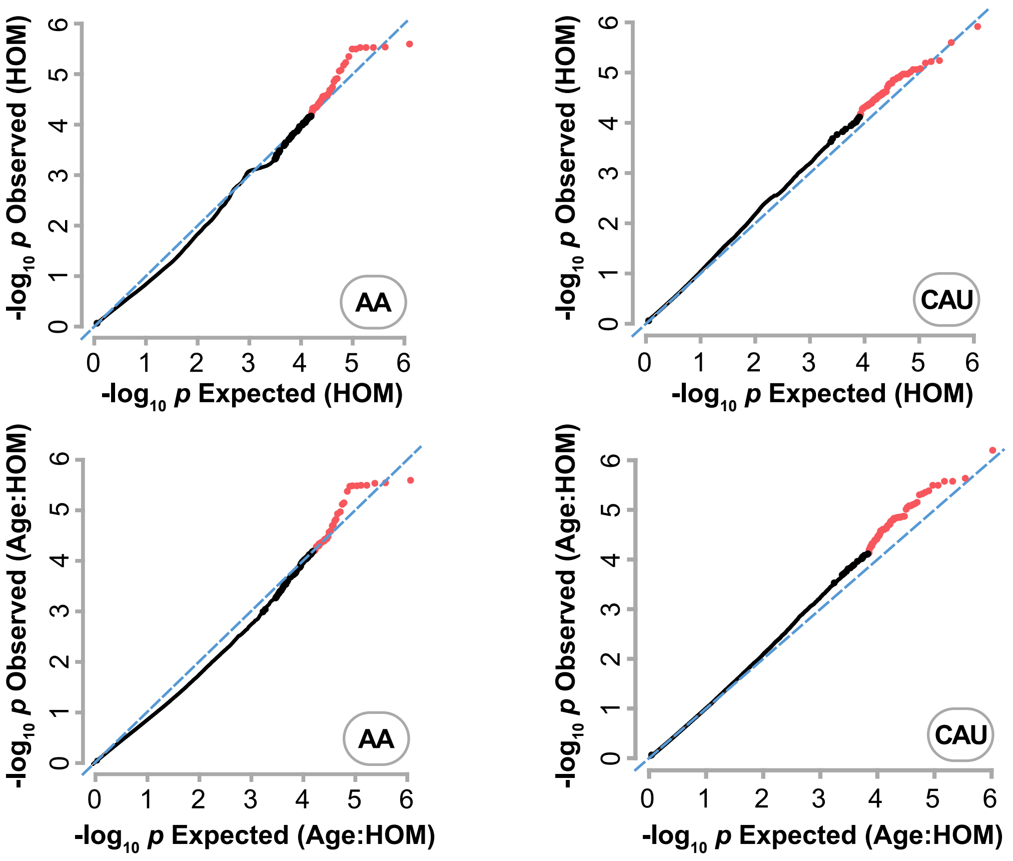

(a) (b)

Figure S4. Q-Q plots of the SNP main effect and SNP-Age interaction effect. The observed versus expected values of the inverse log of the p values (-log10) for the homozygous main effect term (HOM, top) or the SNP-Age interaction term (Age:HOM, bottom) are plotted on the Y or X axis, respectively in the AA **(a)** or CAU **(b)** cohort. *p* values were calculated from equation (2). Red points represent SNPs that associated with ΔSBP at $p > 1E\text{-}4$ and the dashed line represents theoretical distribution of *p* values.

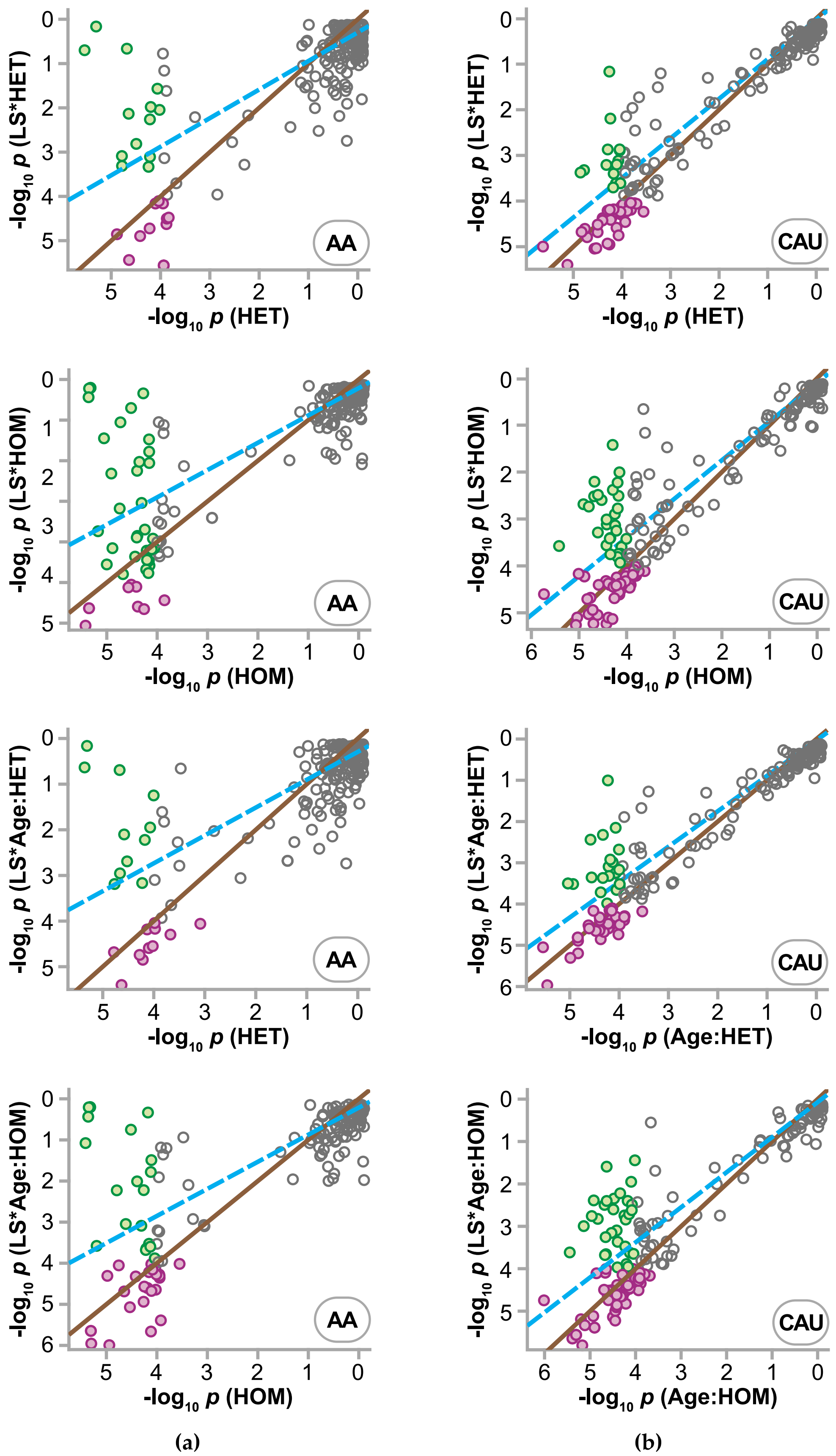

Figure S5. Lifestyle co-participation correction on SNP discovery. The effect of including a lifestyle co-participation variable (LS) in the model for ΔSBP is represented by a scatter plot of the *p* values of the indicated main effect terms (HET, HOM) or interaction terms (Age:HET, Age:HOM) with or without the LS variable on either the Y or X axis, respectively in the AA **(a)** or CAU **(b)** cohort. Regression analysis (dashed line) indicates the overall effect of the correction by how far it deviates from no change (solid

line). Individual SNPs that passed the discovery cutoff of 1E-4 with the LS correction or SNPs that were confounded by LS participation and excluded from additional analyses are indicated by either magenta or green filled points, respectively. Open points represent additional SNPs with no association to ΔSBP (p > 1E-4).
